# *MiR-195* Inhibits Tumor Growth and Metastasis in Papillary Thyroid Carcinoma Cell Lines by Targeting *CCND1* and *FGF2*

**DOI:** 10.1155/2017/6180425

**Published:** 2017-06-27

**Authors:** Yali Yin, Shubin Hong, Shuang Yu, Yanrui Huang, Shuwei Chen, Yujie Liu, Quan Zhang, Yanbing Li, Haipeng Xiao

**Affiliations:** ^1^Department of Endocrinology, The First Affiliated Hospital of Sun Yat-Sen University, Guangzhou 510080, China; ^2^Department of Head and Neck Surgery, Sun Yat-Sen University Cancer Center, Guangzhou 510060, China; ^3^State Key Laboratory of Oncology in South China, Collaborative Innovation Center of Cancer Medicine, Guangzhou, China; ^4^Breast Tumor Center, Sun Yat-Sen Memorial Hospital, Sun Yat-Sen University, Guangzhou 510120, China

## Abstract

**Background:**

MicroRNA (miRNA) dysregulation was commonly seen in papillary thyroid carcinoma (PTC), and *miR-195* was verified to be downregulated in PTC by the large data set analysis from The Cancer Genome Atlas (TCGA). Our study aimed to explore the biological functions and the underlying molecular mechanisms of *miR-195* in PTC.

**Methods:**

The relative expression of *miR-195* and its target genes were assessed by quantitative RT-PCR assay in 38 pairs of PTC and the adjacent thyroid tissues. Assays were performed to evaluate the effect of *miR-195* on the proliferation, migration, and invasion in PTC cell lines. Moreover, we searched for targets of *miR-195* and explored the possible molecular pathway of *miR-195* in PTC.

**Results:**

We found that *miR-195* was downregulated in PTC cell lines and tissues. Overexpression of *miR-195* significantly inhibited cell proliferation, migration, and invasion in K1 and BCPAP cell lines. *CCND1* and *FGF2*, which had inverse correlations with *miR-195* in clinical specimens, were found to be the direct targets of *miR-195*. Furthermore, *miR-195* might be involved in PTC tumorigenesis by suppressing the Wnt/*β*-catenin signaling pathway.

**Conclusions:**

These results highlight an important role of *miR-195* in the initiation and progression of PTC and implicate the potential application of *miR-195* in PTC target therapy.

## 1. Introduction

Thyroid carcinoma, whose incidence has been dramatically rising all over the world in recent decades, represents the most prevalent endocrine malignancy [1]. Papillary thyroid carcinoma (PTC) accounts for about 80 percent of all thyroid cancer cases [2]. PTC is one of the largest incidence-increasing cancers among Chinese women [3]. By 2019, PTC is predicted to be the third most common cancer in women at a cost of $18–$21 billion dollars in the United States [4]. Comprehensive therapy including thyroidectomy, radioactive iodine (RAI), and TSH suppression therapy affords curable treatment with five-year survival rate over 95% before tumor cell dissemination. However, distant metastasis and recurrence still occurred in some subtype of PTC, and the five-year survival rate of advanced PTC is about 59% [5]. Thus, exploring the underlying molecular mechanism is of great importance for improving the prognosis of PTC patients.

MicroRNAs (miRNAs) are a conserved class of endogenous, small noncoding RNAs. These lead to the silencing of their target genes by either degrading mRNA molecules or inhibiting their translation [6]. miRNAs have been involved in various biological events including tumorigenesis and metastasis, implying their crucial role in the pathogenesis of diverse human malignancies. The classic upregulated miRNAs, such as *miR-146b*, *miR-222*, *miR-221*, and *miR-151*, were thought to be involved in the development, especially the metastasis of PTC [7, 8]. Circulating *miR-222* and *miR-146b* levels were found to be associated with PTC recurrence and indicate bad clinical survival [9]. Our recent research suggested that *miR-20b* can modulate MAPK/ERK cascade by suppressing SOS1 and ERK2 and repress cell viability, migration, and invasion in PTC [10]. Taken together, it indicates that dysregulated miRNAs play a crucial role in the pathogenesis of PTC.

The *miR-15* family members, *miR-195* included, are known as tumor suppressors in breast cancer [11], prostate cancer [12], and hepatocellular carcinoma (HCC) [13]. Recently, Cong et al. [14] analyzed the expression of miRNAs and genes in 499 PTC samples and 58 normal thyroid tissues obtained from The Cancer Genome Atlas (TCGA) database and found that *miR-195* was downregulated in PTC compared with normal thyroid tissues. However, the role of *miR-195* in PTC has not been confirmed, and the molecular mechanism of *miR-195* regulation in thyroid carcinoma remains unclear.

In this study, we aim to clarify the biological effects of *miR-195* and to explore the possible targets and the regulatory pathway that *miR-195* might be involved in.

## 2. Materials and Methods

### 2.1. Cell Culture and Clinical Specimens

Human thyroid normal cell line Nthy-ori 3-1 and PTC cell line BCPAP were kindly given by Dr. Haixia Guan (The First Affiliated Hospital of China Medical University, Shenyang, China). K1, another PTC cell line, was purchased from the European Collection of Cell Cultures (ECACC, Salisbury, UK). HEK 293T cell was purchased from the American Type Culture Collection (ATCC, Manassas, USA). Cells were maintained at 37°C in Dulbecco's modified Eagle's medium (DMEM, Invitrogen Technologies, USA) with 10% fetal bovine serum (FBS).

All clinical tissues including PTC and the matched normal thyroid tissues were collected at the First Affiliated Hospital and the Cancer Center of Sun Yat-Sen University (Guangzhou, China) from December 2014 to December 2016. The surgical procedure was performed on all patients, and final diagnoses were based upon pathological examination. All subjects provided informed consent, and the study was approved by the ethics committee of Sun Yat-Sen University.

### 2.2. RNA Extraction and Real-Time Quantitative RT-PCR (RT-qPCR)

Total RNA was isolated from cell lines and patient tissues using TRIzol (Life Technologies, USA) according to the manufacturer's instructions. RNA samples were reverse-transcribed by Prime Script™ RT reagent kit (Takara, Dalian, China). RT-qPCR was performed to evaluate *miR-195* expression on a Light Cycler 480II real-time PCR system (Roche Diagnostics, Switzerland) using SYBR Premix Ex Taq™ (Takara, Dalian, China), with U6 and GAPDH as endogenous control. The sequences of primers used in this study were as follows: *miR-195* forward primer: 5′-TAGCAGCACAGAAATATTGGC-3′; reverse primer: Uni-miR qPCR primer (Takara, Dalian, China), and *U6* forward primer: 5′-ACGCAAATTCGTGAAGCGTT-3′; reverse primer: Uni-miR qPCR primer (Takara, Dalian, China), and Cyclin D1 (*CCND1*) forward primer: 5′-TCCTACTACCGCCTCACA-3′;reverse primer: 5′-ACCTCCTCCTCCTCCTCT-3′, and fibroblast growth factor 2 (*FGF2*) forward primer: 5′-TCAAGCAGAAGAGAGAGGAG-3′; reverse primer: 5′-CCGTAACACATTTAGAAGCC-3′, and *GAPDH* forward primer: 5′-GCACCGTCAAGGCTGAGAAC-3′; reverse primer: 5′-TGGTGAAGACGCCAGTGGA-3′. Relative expression quantification was calculated using the comparative cycle threshold (CT) method (2^−^^∆∆CT^).

### 2.3. Western Blot Assay

Total cellular protein was prepared as follows: cells were lysed in RIPA buffer. Nuclear and cytoplasmic extractions were collected using the Nuclear and Cytoplasmic Extraction Reagents kit (Thermo Fisher Scientific, Rockford, USA). Then, obtained proteins were subjected to 10% SDS-PAGE and transferred onto PVDF membranes (Roche Diagnostics, Switzerland). After blocking with 5% skimmed milk, the membranes were incubated overnight with the following primary antibodies: anti-cyclin D1 (Lab Vision, #RB010P0), anti-FGF2 (Cell Signaling Technology, #3196S), anti-c-Myc (Cell Signaling Technology, #5605), anti-MMP-13 (Abcam, #ab51072), anti-*β*-catenin (Abcam, #ab32572), anti-phospho-*β*-catenin^Ser33/37/Thr41^ (Cell Signaling Technology, #9561), and anti-GAPDH (Santa Cruz, #sc-25778). This was followed by incubation with HRP-conjugated secondary antibodies from CST. Antigen-antibody complexes were visualized using the ECL solution (Thermo Fisher Scientific, Rockford, USA).

### 2.4. Immunohistochemistry (IHC)

Paraffin-embedded tissue sections were deparaffinized and hydrated using xylene and graded alcohol to water. Antigen retrieval was performed by incubation of the tissue sections with boiled sodium citrate buffer (pH 6.0) for 3 min. Endogenous peroxidase activity was quenched with 3% H_2_O_2_. Slides were blocked with 5% BSA to reduce nonspecific binding and then incubated with CCND1 (Lab Vision, #RB010P0) or FGF2 (Cell Signaling Technology, #3196S) primary antibody diluted to a concentration of 1 : 100 overnight at 4°C. After incubation with the secondary antibody (Gene Tech) for 30 min at room temperature, slides were detected with the DAB Enhancer solution (Gene Tech) and counterstained with hematoxylin (MX Biotechnologies). Images were taken by a light microscopy.

### 2.5. RNA Oliogoribonucleotides and Plasmid/Lentivirus Constructs

*MiR-195* mimics and negative control (NC) were purchased from GenePharma (Shanghai, China). The specific sequences are as follows: *miR-195* sense: 5′-UAGCAGCACAGAAAUAUUGGC-3′; antisense: 5′-CAAUAUUUCUGUGCUGCUAUU-3′; NC sense: 5′-UUCUCCGAACGUGUCACGUTT-3′; antisense: 5′-ACGUGACACGUUCGGAGAATT-3′. pcDNA 3.1-*CCND1* and pcDNA 3.1-*FGF2* were purchased from GeneRay (Shanghai, China). The 3′-untranslated regions (3′-UTRs) of *CCND1* and *FGF2* were cloned into the pGL3-basic vector (Promega, USA) with *XbaI* and *PciI* at the downstream of the luciferase gene. To mutate the binding sequence of *miR-195* in the 3′-UTRs, a QuikChange, Site-Directed, Mutagenesis kit (Promega, USA) was used following the instruction. The mature sequence of *miR-195* was amplified and cloned into the lentiviral vector LV3-GFP-puro (GenePharma, Shanghai, China) to generate LV3-*miR-195* cell, and negative control LV3-NC was conducted as the same way.

### 2.6. Cell Transfection

RNA oliogoribonucleotides or plasmids were transfected using Lipofectamine 3000 (Invitrogen, USA) following the manufacturer's protocol. A total of 200 nM of miRNA mimics or 1000 ng plasmid were used for each 6-well plate transfection. BCPAP and K1 cells were infected with recombinant LV3-*miR-195*/LV3-NC lentivirus-transducing units plus polybrene (GenePharma, Shanghai, China). After 48 h of transfection, puromycin was added into the culture cells constantly. Stable transfected cells were obtained after 2–4 weeks.

### 2.7. Luciferase Reporter Assay

HEK 293T cells seeded in 24-well plates were cotransfected with 400 ng of firefly luciferase reporter containing the 3′-UTR (wild-type or mutant) of *CCND1* or *FGF2*, 10 ng of pRL-TK, and 20 pmol of miRNA mimics. Luciferase activities were measured 48 h after transfection using the dual-luciferase reporter assay system (Promega, Madison, USA).

### 2.8. Cell Proliferation and Colony Formation Assays

EdU assay was performed to assess the cell proliferative ability using the EdU kit (Ribobio, Guangzhou, China) following the manufacturer's manuals. For the colony formation assay, after two days of transfection, indicated cells were seeded at 500/well into each 6-well plate and cultured for two weeks. Cell colonies were stained in a dye solution containing 1% crystal violet.

### 2.9. Scratch Wound-Healing Assay

After 48 h of transfection, cells were about 90–95% confluence in 6-well plate. Streaks were carefully scratched with sterile pipette tips. Then, cells were cultured in medium with no serum overnight. The widths of wound were observed and photographed using an inverted microscope (Leica, Germany).

### 2.10. Cell Migration and Invasion Assays

Cells resuspended in 100 *μ*L serum-free medium were plated in the top chamber of each insert (Corning, USA) with a non-Matrigel-coated membrane for the Transwell migration assay and a Matrigel-coated membrane (BD Bioscience, MA, USA) for the invasion assay. Lower chambers of the inserts were filled with 600 *μ*L medium with 10% FBS. After several hours of incubation, cells that invaded to the lower surface of the insert were fixed, stained, and imaged using an DMI4000B inverted microscope (Leica, Germany).

### 2.11. Xenograft Tumor Formation

K1 cells stably infected with the LV3-*miR-195* or LV3-NC were harvested and washed by phosphate-buffer saline. Then, cells (5 × 10^6^) were subcutaneously injected into the right flank of BALB/c nude mice (8 per group). The width and length of tumors were measured every 5 days. Tumor volumes were calculated by the formula: *V* = width^2^ × length/2. On day 23 after implantation, mice were sacrificed and the tumor weights were assessed. The animal study was approved by the Animal Ethical Committee of Sun Yat-Sen University.

### 2.12. Statistical Analysis

We used SPSS software (version 20.0) for all statistical analyses. The significance of different groups of data was calculated with two-tailed Student's *t* test or with one-way ANOVA analysis. All data are presented as the mean ± standard deviation (SD) from at least triple replicates. Spearman's correlation analysis was performed between *miR-195* and its target genes. *P* < 0.05 was considered statistically significant.

## 3. Results

### 3.1. *MiR-195* Is Downregulated in PTC Clinical Specimens and Cell Lines

**Figure 1:**
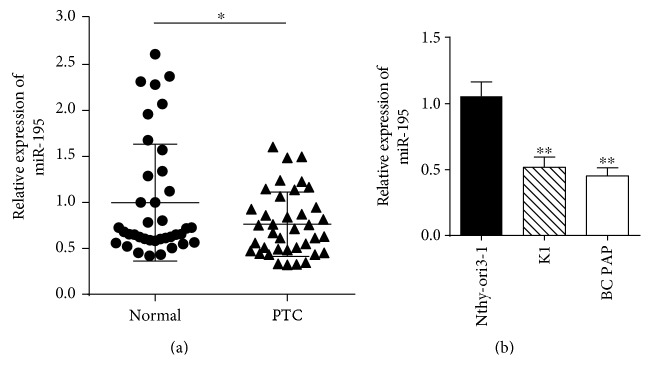
*MiR-195* is downregulated in PTC clinical specimens and cell lines. (a) The relative expression of *miR-195* in PTC tissues compared with matched adjacent normal thyroid tissues by qRT-PCR assay (*n* = 38). (b) *MiR-195* expression in normal thyroid cell line Nthy-ori 3-1 and PTC cell lines K1 and BCPAP. ^∗^*P* < 0.05, compared with the normal group. ^∗∗^*P* < 0.01, compared with Nthy-ori 3-1.

To determine the potential role of *miR-195* in PTC, we analyzed the relative expression of *miR-195* in 38 pairs of PTC tissues and two cell lines. The average expression of *miR-195* was downregulated in PTC tissues compared with the matched normal thyroid tissues (*P* < 0.05, Figure 1(a)). Furthermore, compared with normal thyroid cell line Nthy-ori 3–1, *miR-195* was significantly decreased in K1 and BCPAP cell lines (*P* < 0.01, Figure 1(b)).

We assessed the correlation between miR-195 and clinicopathologic status of PTC patients. The results showed that the level of miR-195 was almost significantly associated with cervical LN metastasis (*P* = 0.07, Supplementary Table 1 available online at https://doi.org/10.1155/2017/6180425). Possible associations between the level of miR-195 and extrathyroidal invasion and TNM stage were analyzed; the data showed a trend that more invasive and advanced stage cancers have lower expression level of miR-195. However, no significant *P* value was found (Supplementary Table 1). This was thought to be caused by the small number of clinical samples.

### 3.2. *MiR-195* Inhibits PTC Cell Growth In Vivo and In Vitro

*MiR-195* mimics was transfected into K1 and BCPAP cell lines. The relative expression of *miR-195* was significantly higher in the mimic group than the control group after transfection (Supplementary Figure 1). To identify the role of *miR-195* in cell growth, EdU assay and colony formation assay were performed.

**Figure 2:**
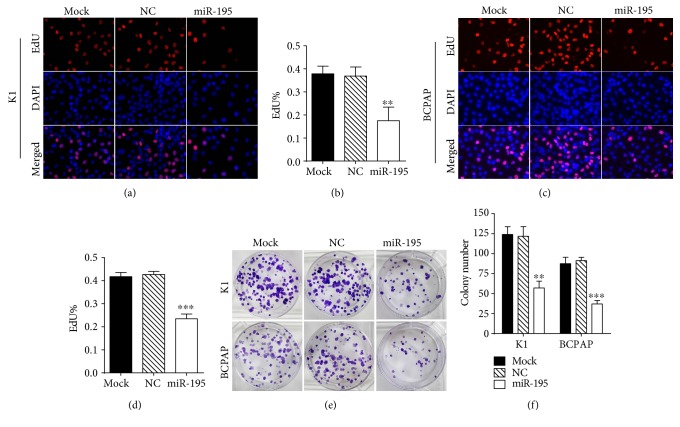
*MiR-195* inhibits PTC cell proliferation in vitro. (a) Fluorescence images of proliferative cells (red) stained with EdU and nuclei (blue) counterstained with DAPI in K1 cells. Magnification, 400x. (b) Quantification of EdU incorporation assay in K1 cells. (c) Fluorescence images of proliferative cells (red) stained with EdU and nuclei (blue) counterstained with DAPI in BCPAP cells. Magnification, 400x. (d) Quantification of EdU incorporation assay in BCPAP cells. (e) Colony formation assay of K1 and BCPAP cells transfected with *miR-195*. (f) Quantification of the colony number in K1 and BCPAP cells. ^∗∗^*P* < 0.01 compared with the control group, ^∗∗∗^*P* < 0.001 compared with the control group.

The EdU assay showed that the number of EdU positive cells was significantly lower in *miR-195* overexpressing cells than control cells (Figures 2(a), 2(b), 2(c), and 2(d)). The colony formation assay confirmed the decrease rate of growth in miR-195 overexpressing cells. It showed that *miR-195* overexpressing PTC cells generated a significantly lower number of colonies as compared with control cells (Figures 2(e) and 2(f)).

**Figure 3:**
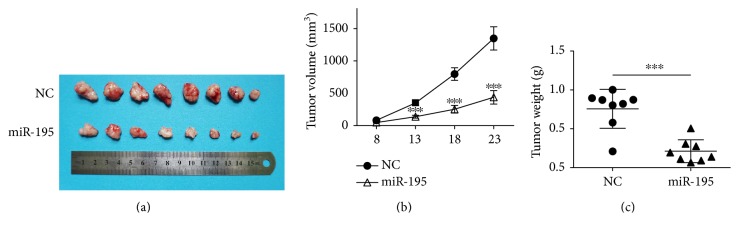
*MiR-195* suppresses xenograft tumor growth of K1 cells in nude mice. (a) Tumors derived from NC or *miR-195* overexpressing K1 cells were dissected from nude mice (*n* = 8) at 23 days after subcutaneous injection. (b) Volume of tumors. (c) Weight of tumors. ^∗∗∗^*P* < 0.001 compared with the NC group.

Additionally, the xenograft tumor formation assay was performed to assess the growth-inhibitory effect of miR-195 in vivo. Intriguingly, we found *miR-195* significantly reduced tumor growth (Figure 3(a)). Both the average tumor volume and the tumor weight were obviously lower in LV3-*miR-195* group mice compared with those in the LV3-NC group (Figures 3(b) and 3(c)). These results indicate that *miR-195* suppressed thyroid tumor growth in vitro and in vivo.

### 3.3. *MiR-195* Suppresses PTC Cell Migration and Invasion

**Figure 4:**
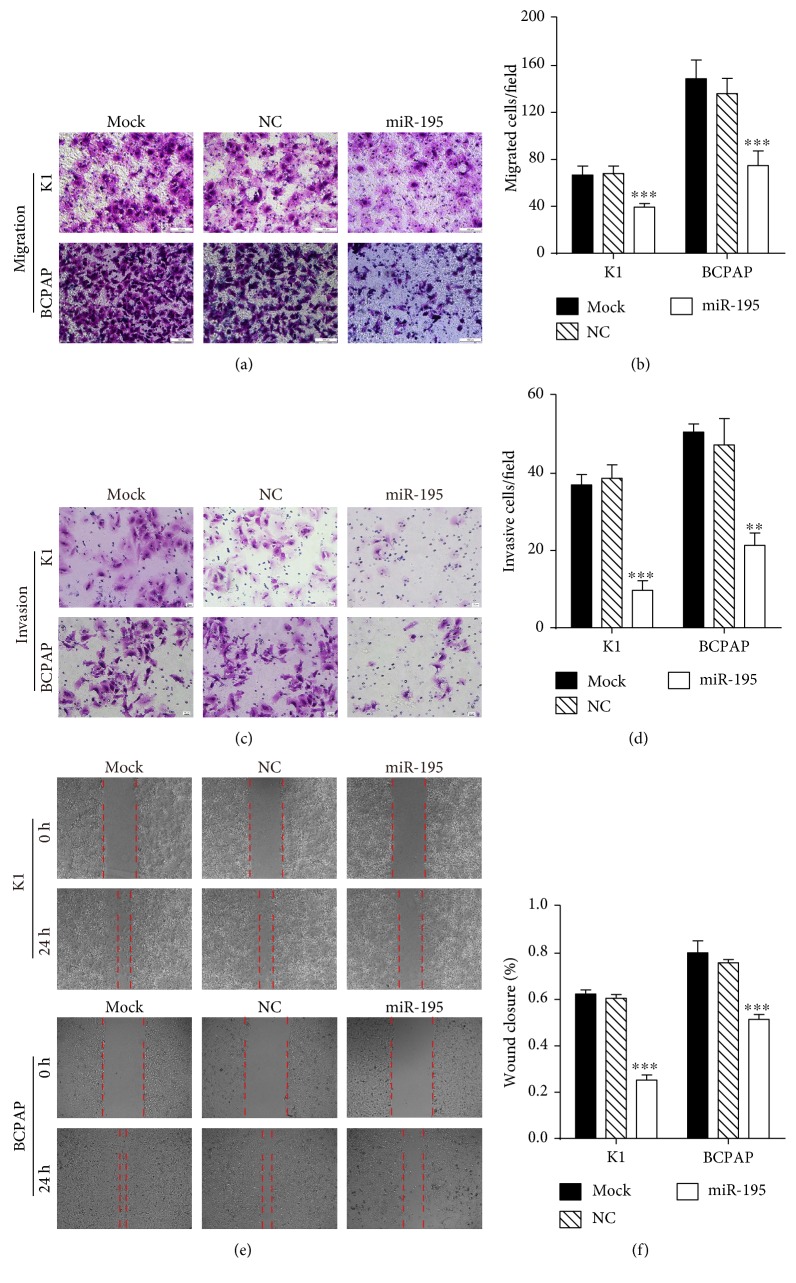
*MiR-195* suppresses PTC cell migration and invasion. (a) Transwell migration assays of K1 and BCPAP cells overexpressing *miR-195* were performed without the Matrigel-coated chamber. Magnification, 200x. (b) Quantification of migrated cells. (c) Transwell invasion assays of K1 and BCPAP cells overexpressing *miR-195* were performed with the Matrigel-coated chamber. Magnification, 200x. (d) Diagrams of invasive cells. (e) Wound healing assays of PTC cells after transfection of *miR-195*. (f) Percentage of wound healing closure assays. ^∗∗^*P* < 0.01 compared with the control group, ^∗∗∗^*P* < 0.001 compared with the control group.

To elucidate the effects of *miR-195* on the migration and invasion of PTC cells, Matrigel-coated or Matrigel-uncoated Transwell assays were analyzed. Both the invasive and migratory activities in K1 and BCPAP cells were suppressed by *miR-195* (Figures 4(a), 4(b), 4(c), and 4(d)). Also, the wound healing assay illustrated that *miR-195* overexpression impaired the wound closure ability of K1 and BCPAP cells (Figures 4(e) and 4(f)). Collectively, these data suggest that *miR-195* inhibited PTC cell migration and invasion.

### 3.4. *MiR-195* Directly Targets the 3′-UTRs of *CCND1* and *FGF2*

**Figure 5:**
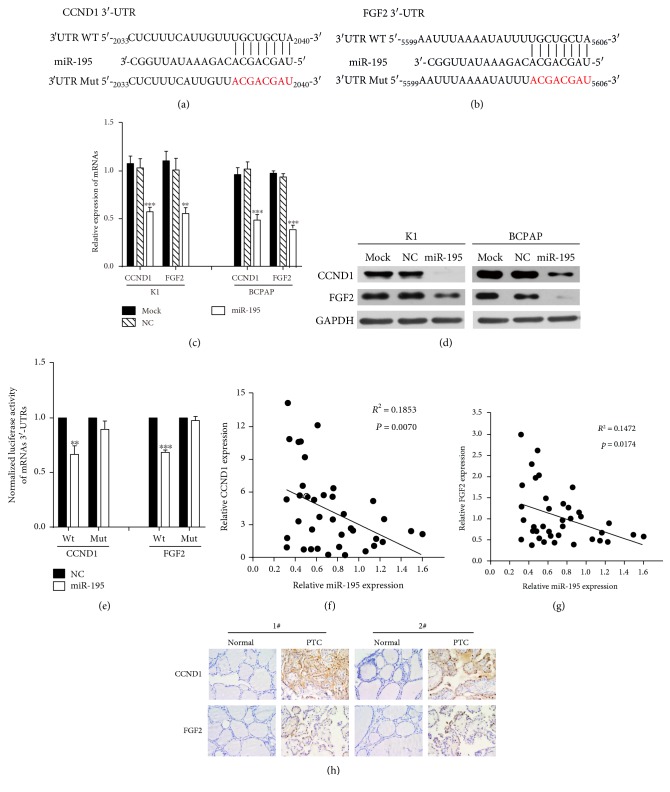
*MiR-195* directly targets the 3′-UTRs of *CCND1* and *FGF2*. (a, b) Predicted binding sites of *miR-195* seed sequence and 3′-UTRs of *CCND1* and *FGF2*. The sequences precisely modified were marked in red. (c) The relative mRNA expression of *CCND1* and *FGF2* in K1 and BCPAP cells 24 h after *miR-195* transfection. (d) Protein levels of CCND1 and FGF2 48 h after *miR-195* transfection. (e) Luciferase assays of *CCND1* and *FGF2* in 293T cell. (f, g) Significant inverse correlations were presented between *miR-195* and *CCND1* as well as *FGF2* in human PTC tissues. (h) IHC detection of CCND1 and FGF2 proteins in normal thyroid and PTC tissues. Two representative examples are shown. Magnification, 400x. ^∗∗^*P* < 0.01 compared with the control group, ^∗∗∗^*P* < 0.001 compared with the control group.

We searched for putative targets of *miR-195* using miRanda (http://www.microrna.org/microrna/home.do) and TargetScan (http://www.targetscan.org/). Among hundreds of promising targets, *CCND1* and *FGF2* were chosen because of their well-known importance in cell growth [15] and metastasis [16], respectively. The predicted binding sites of *miR-195* seed sequence and 3′-UTRs of its target genes are shown in Figures 5(a) and 5(b). The sequences precisely modified were marked in red. The mRNA expression levels of *CCND1* and *FGF2* were significantly decreased with *miR-195* transfection in K1 and BCPAP cells (Figure 5(c)). In the same vein, the protein levels of these two genes were suppressed in *miR-195* overexpressing cells (Figure 5(d)). To confirm the direct relationships between *miR-195* and its target genes, a dual-luciferase reporter assay was performed. It revealed that cotransfection of *miR-195* inhibited the activity of luciferase reporter with wild-type 3′-UTR of *CCND1* and *FGF2*. However, this effect was abrogated when the target site was mutated (Figure 5(e)). Furthermore, we assessed the relative expression of *CCND1* and *FGF2* mRNAs by qRT-PCR in the same set of clinical samples shown in Figure 1(a). Obvious inverse correlations between miR-195 and *CCND1* as well as *FGF2* were confirmed (Figures 5(f) and 5(g)). CCND1 and FGF2 protein expression of the same clinical samples was analyzed by immunohistochemistry staining. As shown in Figure 5(h), intensive CCND1 and FGF2 expression was detected in PTC as compared with normal thyroid tissues.

These data indicate that *miR-195* negatively regulates *CCND1* and *FGF2* expression by directly targeting their 3′-UTRs and this target effect is consistent with the inverse correlations in clinical samples.

### 3.5. Overexpression of *CCND1* or *FGF2* Can Rescue the Inhibitory Function of *miR-195* in PTC Cells

**Figure 6:**
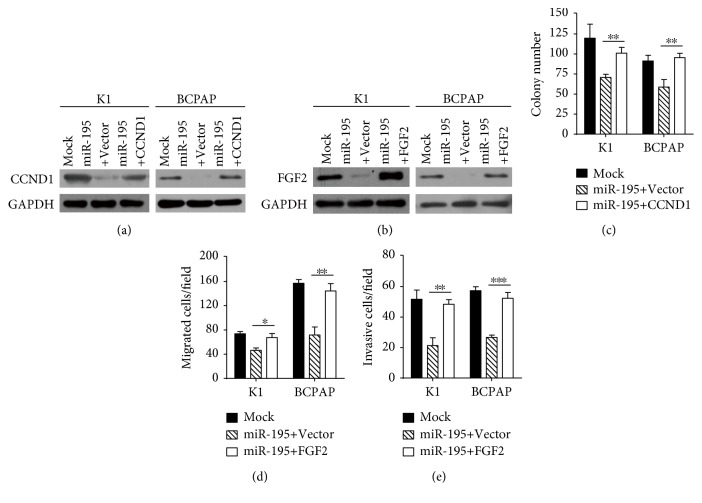
Overexpression of CCND1 or FGF2 can rescue the inhibitory function of miR-195 in PTC cells. (a, b) Ectopic expression of CCND1 and FGF2 were confirmed by Western blot. (c) Colony formation assays show that reexpression of *CCND1* reverses the effect of *miR-195* on PTC cell proliferation. (d, e) Transwell migration and invasion assays indicate reexpression of *FGF2* can antagonize the inhibitory effects of *miR-195* on PTC cell migration and invasion. ^∗^*P* < 0.05 compared with the control group, ^∗∗^*P* < 0.01 compared with the control group, and ^∗∗∗^*P* < 0.001 compared with the control group.

Next, we investigated whether *CCND1* and *FGF2* were functionally related with *miR-195*. K1 and BCPAP cells were cotransfected with *miR-195* and *CCND1* or *FGF2* plasmids (Figures 6(a) and 6(b)). The results showed reexpressing *CCND1* partially abrogated the growth inhibitory effect of *miR-195* (Figure 6(c)). Meanwhile, restored expression of *FGF2* could antagonize the *miR-195* induced inhibition of cell migration and invasion (Figures 6(d) and 6(e)).

As a whole, these findings point that *miR-195* inhibits cell growth by targeting *CCND1* and suppresses migration and invasion by targeting *FGF2* in PTC cells.

### 3.6. *MiR-195* Suppresses the Wnt/*β*-Catenin Pathway in PTC Cells and Xenograft Tumors

**Figure 7:**
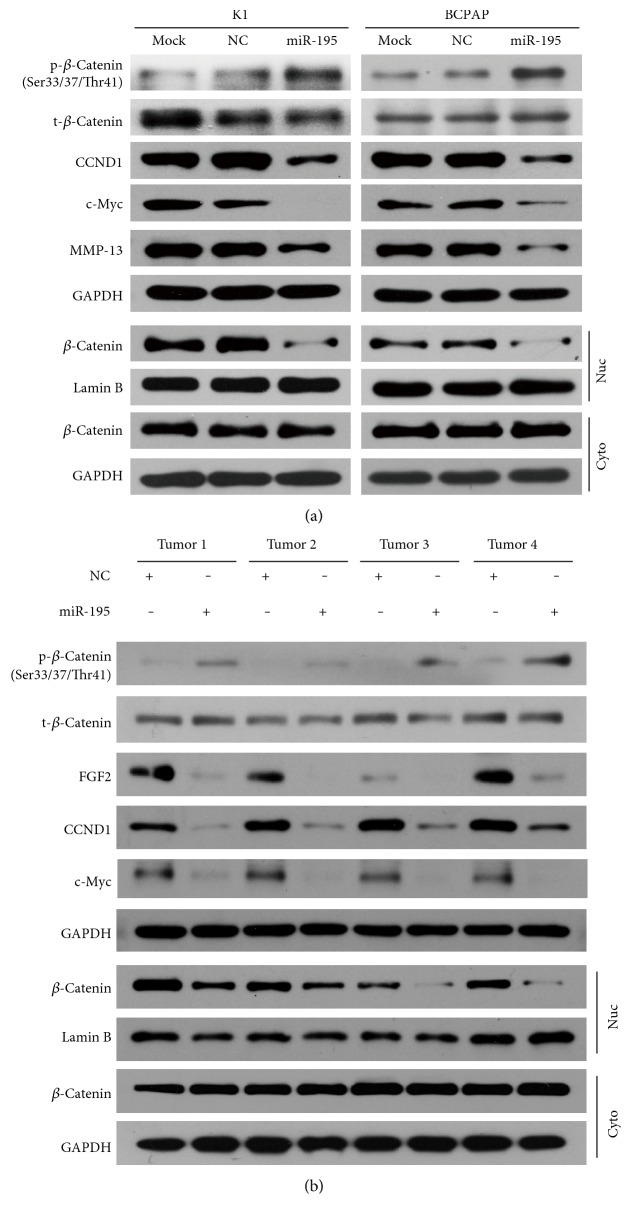
MiR-195 suppresses the Wnt/*β*-catenin pathway in PTC cells and xenograft tumors. (a) Western blotting analysis of proteins involved in Wnt/*β*-catenin pathway and MMP13 in PTC cells. The phosphorylation of *β*-catenin at Ser33/37/Thr41 sites increased in *miR-195* overexpressing K1 and BCPAP cells. CCND1, c-Myc, MMP13 proteins, and nuclear *β*-catenin declined while cytoplasmic *β*-catenin almost unchanged after *miR-195* transfection. (b) Expression profile changes of CCND1, FGF2, and proteins involved in Wnt/*β*-catenin pathways in xenograft tumor tissues. The miR-195 overexpression group demonstrated increased phos-*β*-catenin and decreased nuclear *β*-catenin. The expression levels of CCND1, FGF2, and c-Myc proteins were significantly reduced in the miR-195 group compared with the NC group. Tumors 1, 2, 3, and 4 are from four different mice. Lamin B and GAPDH presented as control. Nuc: nuclear; Cyto: cytoplasmic.

As the Wnt/*β*-catenin cascade is of great importance in PTC pathogenesis, we investigated the effects of *miR-195* on this signaling pathway. *MiR-195* overexpressing remarkably increased the phosphorylation of *β*-catenin (Figure 7(a)). To trace the amount of *β*-catenin translocating into the nucleus, which is the effective factor transcribing various tumor-promoting genes, we separated nuclear proteins from cytoplasmic ones and found upregulation of *miR-195* caused a decline of nuclear *β*-catenin (Figure 7(a)). Moreover, the downstream protein of the Wnt/*β*-catenin pathway, namely, c-Myc, was obviously suppressed (Figure 7(a)). Besides, as *FGF2* is an in vivo modulator of matrix metallopeptidase 13 (MMP-13) expression in malignant tumors [17], a significant decrease in the level of MMP-13 protein was also observed in *miR-195* overexpressing cells (Figure 7(a)). We also collected proteins from the xenograft in mice and detected the expression level of targets as well as proteins involved in Wnt/*β*-catenin pathways. As expected, miR-195 overexpression group demonstrated increased phos-*β*-catenin and decreased nuclear *β*-catenin. The expression levels of CCND1, FGF2, and c-Myc proteins were significantly reduced in the miR-195 group compared with the NC group (Figure 7(b)).

Taken together, these data indicate that *miR-195* could influence the Wnt/*β*-catenin signaling pathway and MMP 13 in PTC cells.

## 4. Discussion

miRNAs and protein-coding RNAs consist of a complicated network, which modulates the initiation and progression of cancers including PTC. In this network, numbers of miRNAs such as *miR-146b* [18], *miR-222* [19], and *miR-20b* [10] have been proven to promote or suppress the progression of PTC. However, there are still numerous miRNAs whose biological functions and molecular mechanisms remain unknown. Our current study showed that the relative expression of *miR-195* was significantly decreased in PTC tissues and cell lines, and it suppressed proliferation, migration, and invasion in PTC cells. The antiproliferative function of *miR-195* in vivo was demonstrated by xenograft tumor formation experiment.

It was found that m*iR-195* was reduced in HCC and exerted a role in the tumorigenesis of HCC [20]. In prostate cancer, m*iR-195* inhibited cell epithelial-mesenchymal transition (EMT) [21]. Moreover, m*iR-195* exerted its tumor suppressive effect by targeting *VEGF* [22], *IKKα*, and *TAB3* [13] in HCC. *MiR-195* overexpressing enhanced the radiosensitivity of breast cancer by targeting *BCL-2* [23]. In the present study, we found *CCND1* and *FGF2* were functional targets of *miR-195* in PTC and miR-195 was inversely correlated with these two targets.

*CCND1*, one of the highly conserved members of the cyclin family, was characterized by a periodicity in protein abundance throughout the cell cycle. The deregulation of *CCND1* expression was regarded as a hallmark of cancer by causing continuous abnormal proliferation, thus playing as an oncogene [15, 24]. Overexpression of *CCND1* gene was observed in both benign and malignant thyroid tumors [25]. In addition, it was found that *CCND1* has been regulated by multitudinous miRNAs. For example, *miR-138* inhibited nasopharyngeal carcinoma growth by targeting *CCND1* oncogene [26]. Cai et al. found *CCND1*, *CDK2*, and *CDK6* directly targeted by *miR-186* in lung adenocarcinoma [27]. Our results showed that restoration of *miR-195* induced PTC cell growth arrest by targeting *CCND1* in vivo and in vitro.

Fibroblast growth factor 2 (*FGF2*), a member of the *FGF* family, controls various cellular processes in different contexts, including migration and invasion [28]. *FGF2* is highly expressed in differentiated thyroid cancer [29], as well as in many other malignancies including breast cancer [30] and HCC [31], implying its important role in tumorigenesis. Afterwards, *FGF2* was proven to be associated with lymph node invasion and distant metastasis in differentiated thyroid cancers [32, 33]. It suggests that *FGF2* plays an important role in thyroid cancer progression. Furthermore, the molecular mechanisms of *FGF2* involving oncogenesis were gradually clarified. *MiR-503* inhibited tumor angiogenesis in HCC by targeting *FGF2* and *VEGFA* [34]. Besides, *miR-646* was found to suppress osteosarcoma cell metastasis by downregulating *FGF2* [35]. Meanwhile, *FGF2* has been reported to regulate MMP-13 expression with a time- and dose-dependent relation in chondrosarcoma cells [17], leading to the degradation of extracellular migration inhibitory factor (MIF) to promote cancer metastasis [36]. Upregulation of MMP-13 was significantly related with TNM stage and recurrent disease in PTC [37]. Our current study showed that *miR-195* inhibited cell migration and invasion by targeting *FGF2* and the con-transfection of *miR-195* and *FGF2* plasmid which can abrogate the inhibitory effect. The possible mechanism of anti-invasive effect derives from the decreasing level of the downstream MMP-13 protein. Taken together, *miR-195*-*FGF2*-MMP-13 axis may be a new target for thyroid cancer metastasis.

The Wnt pathway, especially the canonical Wnt signaling, is involved in many developmental and physiological processes [38], especially in human cancer [39]. Wnt activation induced *β*-catenin stabilization and nuclear accumulation, leading DNA-bound transcription factor TCF to complex with *β*-catenin. Together with some other coactivator, this complex activates target genes such as *CCND1* and *c-Myc* [40]. Whereas Wnt signaling restraining can result in *β*-catenin phosphorylation at serines 33 and 37 and threonine 41, leading to *β*-catenin ubiquitination and degradation [41]. Activation of the Wnt/*β*-catenin signaling is often caused by activating mutations of *CTNNB1* (which encodes *β*-catenin) in thyroid cancer, particularly in poorly differentiated thyroid cancer (PDTC) and anaplastic thyroid carcinoma (ATC) [42, 43]. Delocalization of *β*-catenin was reported to significantly correlate with upregulation of *CCND1* in PTC tissues, suggesting the Wnt/*β*-catenin signaling involved in PTC tumorigenesis [44]. The *miR-15/16* cluster was found to decrease the expression of *β*-catenin by targeting *WNT3A* signaling in prostate cancer [45]. Whether *miR-195* has a similar molecular mechanism as the same family member does remains unknown. Our data demonstrated that *miR-195* overexpression increased *β*-catenin phosphorylation, decreased nuclear *β*-catenin amount, and thus, suppressed the Wnt/*β*-catenin signaling in PTC.

With the crucial functions of the Wnt signaling in cancerous transformation and growth, the need for specific drugs targeting the Wnt pathway is urgent. Vandetanib, selectively targeting RET, VEGFR, and EGFR tyrosine kinases, has been approved for the treatment of medullary thyroid carcinoma (MTC). More than that, it was found to inhibit cell growth and migration in PTC by stabilizing *β*-catenin and decreasing the downstream target genes *c-Myc* and *CCND1* [46]. Meanwhile, some traditional nonsteroidal anti-inflammatory drugs (NSAIDs), such as aspirin, showed an interesting anticancer effect by inhibiting the Wnt signaling [47]. The current study showed the obvious evidence of *miR-195* for suppressing the Wnt/*β*-catenin signaling, indicating the potential target for thyroid cancer therapy. The prospective clinical utility of miR-195 deserves further study.

## 5. Conclusions

In summary, downregulation of *miR-195* is demonstrated in PTC. *MiR-195* exerts its tumor suppressive function by targeting *CCND1* and *FGF2* and restraining the activity of the Wnt/*β*-catenin signaling. Our findings suggest an important role of *miR-195* in the pathogenesis of PTC, and miR-195-FGF2-MMP-13 axis might be a potential new target for PTC.

## Supplementary Material

Supplementary Figure 1. The relative expression of miR-195 in mimics transfected cells is significantly higher than control cells. ^∗∗^*P* < 0.01, compared with control group. Supplementary Table 1. Clinicopathologic features of PTC with associated miR-195 relative expression.
