# Supplementary material for: MiR-195 Inhibits Tumor Growth and Metastasis in Papillary Thyroid Carcinoma Cell Lines by Targeting CCND1 and FGF2
Source: Int J Endocrinol. 2017 Jun 27;2017:6180425. doi: 10.1155/2017/6180425 (PMC5504932; doi:10.1155/2017/6180425)
Supplement: Supplementary file 1 — Supplementary Figure 1. The relative expression of miR-195 in mimics transfected cells is significantly higher than control cells. ∗∗P < 0.01, compared with control group. Supplementary Table 1. Clinicopathologic features of PTC with associated miR-195 relative expression. [file 6180425.f1.pdf]

Article title: MiR-195 inhibits tumor growth and metastasis in papillary thyroid carcinoma cell lines by targeting CCND1 and FGF2

Journal name: International Journal of Endocrinology

Author names:

Yali Yin[1]*, Shubin Hong[1]*, Shuang Yu[1], Yanrui Huang[1], Shuwei Chen[2,3], Yujie Liu[4], Quan Zhang[2], Yanbing Li[1], Haipeng Xiao [1 #]

Institutional addresses:

[1]Department of Endocrinology, The First Affiliated Hospital of Sun Yat-Sen University, Guangzhou, 510080, China.

[2]Department of Head and Neck Surgery, Sun Yat-Sen University Cancer Center, Guangzhou, 510060, China.

[3]State Key Laboratory of Oncology in South China, Collaborative Innovation Center of Cancer Medicine, Guangzhou, China.

[4]Breast Tumor Center, Sun Yat-Sen Memorial Hospital, Sun Yat-Sen University, Guangzhou, 510120, China.

Author E-mail:

Yali Yin: smart_yyl@163.com; Shubin Hong: robin151208@sina.com; Shuang Yu: shuang.yu@aliyun.com; Yanrui Huang: you9251@163.com; Shuwei Chen: chenshuw@sysucc.org.cn; Yujie Liu: liuyujie554@163.com; Quan Zhang: zhangquan@sysucc.org.cn; Yanbing Li: easd04lyb@126.com; Haipeng Xiao: xiaohp@mail.sysu.edu.cn

*These authors have contributed equally to this work.

[#] Correspondence: Haipeng Xiao, xiaohp@mail.sysu.edu.cn, T: 86(20)87755776-8803.

Running title : Downregulation of *miR-195* in papillary thyroid carcinoma.

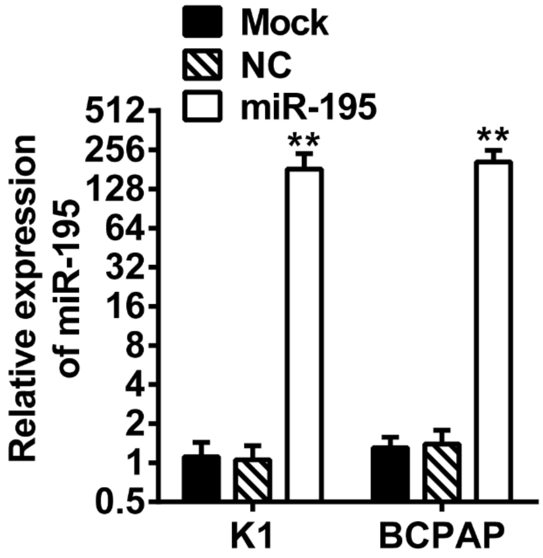

Supplementary Fig. 1. The relative expression of *miR-195* in mimics transfected cells is significantly higher than control cells. ** $P < 0.01$, compared with control group.

Supplementary Table 1. Clinicopathologic features of PTC with associated miR-195 relative expression

Clinicopathologic features	n	*miR-195*	*P* value
Age, years			
<45	28	0.69±0.31	0.33
≥45	10	0.80±0.38	
Sex			
Male	12	0.73±0.35	0.86
Female	26	0.71±0.32	
Tumor size, cm			
<2	20	0.70±0.33	0.79
≥2	18	0.73±0.34	
Multicentricity			
No	25	0.73±0.33	0.80
Yes	13	0.70±0.34	
Extrathyroidal invasion			
No	15	0.78±0.38	0.14
Yes	23	0.63±0.21	
Cervical LN metastasis			
No	11	0.87±0.36	0.07
Yes	27	0.66±0.28	
TNM stage			
I/II	28	0.81±0.38	0.33
III/IV	10	0.69±0.31	

PTC, papillary thyroid carcinoma; LN, lymph node; TNM, tumor, node, metastasis, classification.
